# Stereophotogrammetry to reveal age‐related changes of labial morphology among Chinese women aging from 20 to 60

**DOI:** 10.1111/srt.12906

**Published:** 2020-06-26

**Authors:** Yuming Chong, Ruijia Dong, Xinyu Liu, Xiaojun Wang, Nanze Yu, Xiao Long

**Affiliations:** ^1^ Chinese Academy of Medical Sciences & Peking Union Medical College Beijing People's Republic of China; ^2^ Department of Plastic and Aesthetic Surgery Peking Union Medical College Hospital Chinese Academy of Medical Sciences & Peking Union Medical College Beijing People's Republic of China

**Keywords:** 3D photography, aging, anthropometric, Chinese women, labial morphology

## Abstract

**Background:**

The lip is of important aesthetic value and highly subjected to aging. Collecting anthropometric baseline data and understanding age‐related changes of labial morphology can help with diagnosis of deformity, assessment of aging, and planning of cosmetic procedures. Many studies have focused on Caucasians, while there is a lack of anthropometric data on Chinese women.

**Methods:**

A total of 169 women were enrolled in this cross‐sectional study and divided into four consecutive age groups. Linear distances, angles, and surface area data were obtained via stereophotogrammetry. Intergroup comparisons between different age groups were performed to find age‐related differences.

**Results:**

Lip width significantly increased with age while philtrum width seemed to show no obvious change. Cutaneous upper and lower lip height increased, lengthening the lip in the vertical dimension. Decrease of upper vermilion height and changes in angles indicated that aging process shortened the upper vermilion and flattened the vermilion border. Surface area also showed age‐related changes. Intergroup comparison showed no statistical significance in most variables between 20s and 30s or 30s and 40s, while age‐related changes in some variables were significant between 40s and 50s.

**Conclusion:**

This study provided anthropometric data of labial morphology across a wide age range. Aging process affected a variety of labial anthropometric variables. Age‐related changes accelerated after 40 among Chinese women.

## 1 INTRODUCTION

Pursuit of youthful appearance has brought increasing demand for cosmetic procedures to fight against aging.^1^ The lip, holding the key aesthetic features of the lower third of the face, is heavily subjected to aging process.^2^, ^3^ Previous anthropometric studies have revealed vertical lengthening of cutaneous part of upper and lower lip, decreased height of upper and lower vermilion, and upper lip elongation.^4^, ^5^, ^6^ Histological analysis provided evidence of vessel reduction, cutis thinning, and orbicularis oris muscle thinning in aging lips.^7^, ^8^ Magnetic resonance imaging (MRI) suggested upper lip complex experienced a combination of soft‐tissue lengthening and volume loss.^9^

These age‐related changes are often reversed by surgical rejuvenation, usually a combination of a precise lip left and upper lip augmentation.^10^ Before these procedures being practiced, a standard of youthful lip should be set and how much to correct should be known. Many studies have given their answers in Caucasians while there is a lack of data on Chinese women.^11^ It has been pointed out that cosmetic surgery on Asians relying on Western standards might lead to inharmonious facial proportions and a loss of ethnic features, so data depicting lip aging on Asians should be collected.^12^ Since it takes too long to track one subject to observe age‐related changes, cross‐sectional study is a common choice that efficiently provides normative data.^13^ However, many such studies only compared two age groups (usually young subjects in their 20s and elder subjects around 60), while few analyzed several consecutive age groups. The authors believe it is of importance to study a large consecutive age range to give more comprehensive anthropometric data to guide cosmetic procedures.

This study is designed to provide baseline anthropometric data on labial morphology and analyze age‐related morphological changes of the lip of Chinese women . Normative results could help with diagnose of deformity, preoperative assessment, and planning of cosmetic procedures.

## 2 MATERIALS AND METHODS

### 2.1 Study sample

This cross‐sectional study was approved by Institutional Review Board of Peking Union Medical College Hospital. A total of 169 subjects were voluntarily recruited from 12 provinces of Northern China during November 2016 to June 2017 under the following inclusion criteria: (a) Han ethnicity; (b) age between 20 and 60 years old; (c) body mass index between 18.5 and 24.9; (d) class I skeletal pattern; (e) normal occlusion; (f) no craniofacial deformity. Subjects with malocclusion or having craniofacial surgery or orthodontic treatment were excluded.

### 2.2 Data collection

The subjects sat still in natural head position with neutral expression and mouth gently closed. VECTRA H1‐270 camera (Canfield Scientific Inc) was operated by one of the authors strictly according to instructions to capture 3D images under the same brightness. Three shots were taken from right oblique, front, and left oblique views. Three images were automatically stitched into a 3D facial image to allow further modification.

**FIGURE 1:**
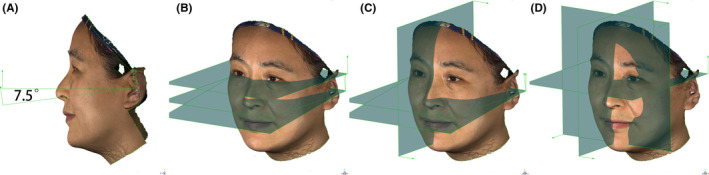
Establishment of a coordinate system. (A) The true horizontal plane was set by rotating the Camper's plane upward for 7.5 degrees; (B) The horizontal plane was set to pass the origin and to be parallel to the true horizontal plane; (C) The sagittal plane was perpendicular to both Camper's plane and true horizontal plane; (D) The coronal plane was perpendicular to both horizontal plane and sagittal plane

As shown in Table 1, anthropometric landmarks were manually identified into each 3D facial images according to the definition proposed by Farkas et al^14^ A coordinate system was built to allow computation of linear distances and angular measurements. The origin of this coordinate system was set as the midpoint of left and right endocanthion. The Camper's plane was defined as the plane passing the right tragus, the left tragus, and the midpoint of right and left alar. The true horizontal plane was obtained by rotating the Camper's plane by 7.5 degrees upward (Figure 1A). The horizontal plane was set to pass the origin and parallel to the true horizontal plane (Figure 1B). The sagittal plane was defined as the plane perpendicular to both Camper's plane and true horizontal plane (Figure 1C). Finally, the coronal plane was set to pass the origin and to be perpendicular to both horizontal plane and sagittal plane (Figure 1D).

**TABLE 1:** Anthropometric landmarks in orolabial region

Abbr.	Landmark	Definition
sn	Subnasale	Midline point at the junction of the lower border of nasal septum and the superior border of upper lip
cphR	Crista philtra right	The junction of vermilion and right lateral philtrum crest
cphL	Crista philtra left	The junction of vermilion and left lateral philtrum crest
ls	Labrale superius	Midline point of upper vermilion border
chR	Cheilion right	Midline point at the right end of labial fissure
chL	Cheilion left	Midline point at the left end of labial fissure
sto	Stomion	Midline point at the labial fissure
li	Labrale inferius	Midline point of lower vermilion border
sl	Sublabial	The most posterior midline point at the labiomental region

**FIGURE 2:**
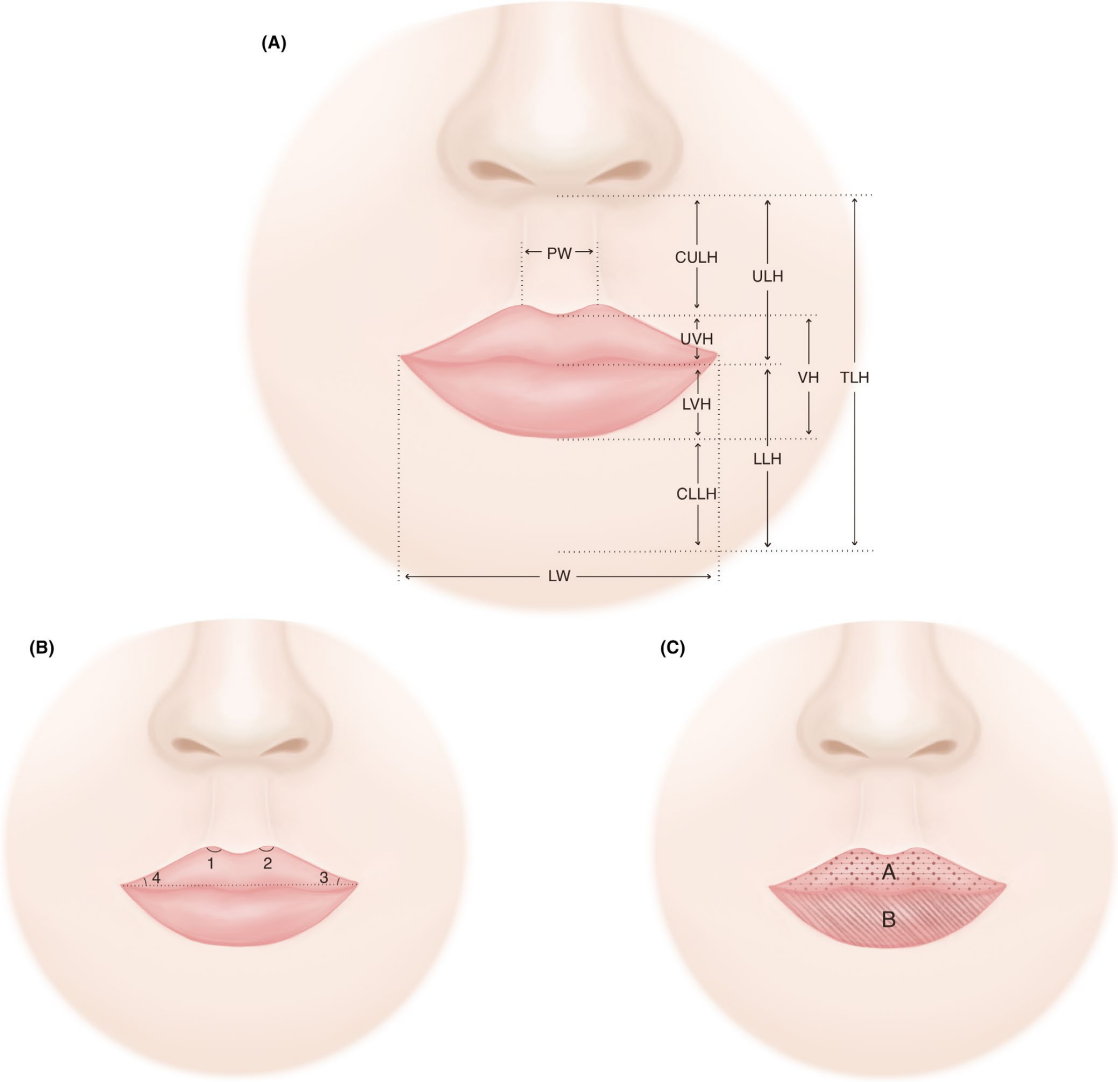
Anthropometric variables. (A) Linear distance variables. CLLH, cutaneous lower lip height; CULH, cutaneous upper lip height; LLH, lower lip height; LVH, lower vermilion height; LW, lip width; PW, philtrum width; TLH, total lip height; ULH, upper lip height; UVH, upper vermilion height; VH, vermilion height; (B) Angular variables; (C) upper and lower lip surface area

Ten linear measurements (Figure 2A) and four angular measurements (Figure 2B) were computed by the coordinates of anthropometric landmarks. Linear variables included philtrum width (PW), lip width (LW), upper lip height (ULH), cutaneous upper lip height (CULH), upper vermilion height (UVH), lower lip height (LLH), cutaneous lower lip height (CLLH), lower vermilion height (LVH), vermilion height (VH), and total lip height (TLH). For each subject, the surface areas of upper and lower lip were separated measured and added up for total lip surface area (Figure 2C). Six proportions, including philtral‐labial score (PLS, computed as the ratio of CULH to UVH), LVH/CLLH, LLH/ULH, PW/LW, ULH/LW, and VH/LW, in the form of ratio or percentage, were evaluated to provide additional anthropometric information.

### 2.3 Method error

To evaluate method error, intra‐observer and inter‐observer reliability were evaluated by intra‐class correlation coefficient (ICC). After the landmark identification, ten 3D photographs were randomly selected from the database for reliability test. Landmarks onto these photographs were removed. Two weeks later, the main observer and an assistant observer identified landmarks onto them independently. All measurements were compared for intra‐ and inter‐observer reliability.

### 2.4 Statistical analysis

Linear and angular measurements are presented as mean ± standard deviation (SD). For each variable, Tukey's post hoc test was performed to analyze difference between two age groups if ANOVA or Kruskal‐Wallis test revealed statistical significance among four groups. Statistical analysis was conducted using SPSS 23.0 (IBM). *P* value < 0.05 was considered statistically significant.

## 3 RESULTS

The study population was divided into four age groups at 10‐year interval. There were 48 women in their 20s, 43 in their 30s, 43 in their 40s, and 35 in their 50s. The average age of four age groups was 24.58 ± 2.68, 34.40 ± 3.15, 44.95 ± 2.56, and 55.97 ± 4.19, respectively.

Table 2 demonstrated the results of intra‐ and inter‐observer reliability. Intra‐class correlation of all anthropometric variables for both intra‐ and inter‐observer reliability ranged from 0.80 to 1.00, indicating excellent reliability of all measurement. The mean difference of all linear measurements was <1 mm, and that of angular measurement was <3 degrees. The mean difference of surface area measurement was also acceptable as it took up a very small percent of the original value. Mean difference for all variables indicated good measurement accuracy.

**TABLE 2:** Intra‐ and inter‐observer reliability

	Intra‐observer reliability		Inter‐observer reliability	
	ICC	Mean difference (mm)	ICC	Mean difference (mm)
Linear measurements				
Philtrum width	0.929	0.08	0.824	−0.05
lip width	0.992	−0.72	0.863	−1.56
Upper lip height	0.983	0.15	0.929	0.11
Cutaneous upper lip height	0.952	0.37	0.959	0.19
Upper vermillion height	0.925	−0.22	0.972	−0.09
Lower lip height	0.994	−0.25	0.976	−0.03
Lower vermillion height	0.97	0.01	0.928	−0.19
Cutaneous lower lip height	0.986	−0.26	0.963	0.16
Vermillion height	0.99	−0.10	0.935	0.07
Total lip height	0.984	−0.21	0.995	−0.28
Angular measurements				
Angle 1	0.871	−2.45	0.917	−1.90
Angle 2	0.843	−2.60	0.952	−1.26
Angle 3	0.967	1.07	0.966	1.27
Angle 4	0.955	1.37	0.991	2.19
Surface area measurements				
Upper lip area	0.953	1.50	0.894	2.63
Lower lip area	0.974	−12.25	0.902	−13.38
Total lip area	0.966	−10.75	0.893	−10.75

Note

Mean difference of linear measurements is in mm; mean difference of angular measurements is in degrees; and mean difference of surface area measurements is in mm^2.^

Table 3 summarized the average measurement of each anthropometric variable of four age groups. Mean difference of all variables between different age groups was shown in Table 4. Philtrum width showed no statistical significance (*P* = .218) among four age groups while month width increased significantly with age, rising from 45.38 ± 2.93 among 20s to 49.51 ± 2.89 among 50s. Upper lip height manifested age‐related increment (*P* = .002) due to the elongation of cutaneous upper lip (*P* < .001), while upper vermilion significantly decreased with age (*P* < .001). Similar age‐related changes were observed in lower lip. Elongation of cutaneous lower lip along with thinning of lower vermilion resulted in increasing lower lip height, though not statistically significant (*P* = .115).

**TABLE 3:** Anthropometric results of four age groups

	20‐29		30‐39		40‐49		50‐59	
	Mean	SD	Mean	SD	Mean	SD	Mean	SD
Linear measurements (mm)								
Philtrum width	11.71	1.35	11.63	1.59	11.22	1.70	12.02	2.14
Lip width	45.38	2.93	47.67	4.16	49.15	3.42	49.51	2.89
Upper lip height	21.85	2.09	22.01	1.66	22.80	1.94	23.31	1.79
Cutaneous upper lip height	13.75	1.82	14.48	1.81	15.37	1.99	16.74	1.66
Upper vermillion height	8.10	1.40	7.53	1.07	7.43	1.35	6.57	1.38
Lower lip height	17.33	1.94	17.88	1.72	17.77	2.57	18.50	2.24
Lower vermillion height	9.79	1.56	9.70	1.61	9.00	1.69	8.57	1.62
Cutaneous lower lip height	7.71	2.50	8.18	1.97	8.77	2.34	9.93	2.64
Vermillion height	17.90	2.59	17.23	2.32	16.43	2.68	15.13	2.58
Total lip height	39.18	3.25	39.88	2.69	40.57	3.52	41.80	3.40
Angular measurements (degrees)								
Angle 1	130.22	7.49	129.38	8.20	132.71	7.94	138.48	8.11
Angle 2	130.04	7.07	127.75	8.40	129.80	8.32	137.41	9.07
Angle 3	32.74	5.74	32.38	5.48	28.80	5.11	25.18	5.58
Angle 4	33.46	5.50	31.32	5.79	28.71	5.04	24.20	6.66
Surface area measurements (mm^2^)								
Upper lip area	478.44	80.94	441.35	67.40	433.14	66.35	399.37	74.13
Lower lip area	467.51	74.91	441.26	82.51	403.86	87.34	381.77	73.83
Total lip area	945.95	144.08	882.60	138.69	837.00	134.90	781.14	130.18

**TABLE 4:** Intergroup comparison and mean difference between different age groups

	ANOVA	Mean difference					
		20‐29 vs 30‐39	20‐29 vs 40‐49	20‐29 vs 50‐59	30‐39 vs 40‐49	30‐39 vs 50‐59	40‐49 vs 50‐59
Linear measurements							
Philtrum width	N/S						
Lipwidth	<0.001	−2.29*	−3.77*	−4.13*	−1.48	−1.84	−0.36
Upper lip height	0.002	0.16	0.95	−1.46*	−0.79	−1.30*	−0.50
Cutaneous upper lip height	<0.001	−0.73	−1.63*	−2.99*	−0.90	−2.26*	−1.37*
Upper vermillion height	<0.001	0.57	0.67	1.54*	0.10	0.97*	0.86*
Lower lip height	N/S						
Lower vermillion height	0.002	0.10	0.79	1.23*	0.70	1.13*	0.43
Cutaneous lower lip height	<0.001	−0.47	−1.06	−2.22*	−0.59	−1.75*	−1.16
Vermillion height	<0.001	0.67	1.47*	2.76*	0.80	2.09*	1.30
Total lip height	0.003	−0.7	−1.39	−2.62*	−0.69	−1.92*	−1.23
Angular measurements							
Angle 1	<0.001	−0.84	−2.49	−8.27*	−3.33	−9.11*	−5.78*
Angle 2	<0.001	2.29	0.24	−7.37*	−2.05	−9.66*	−7.60*
Angle 3	<0.001	0.36	3.94*	7.56*	3.58*	7.20*	3.62*
Angle 4	<0.001	2.13	4.75*	9.26*	2.62	7.13*	4.51*
Surface area measurements							
Upper lip area	<0.001	37.09	45.30*	79.07*	8.21	41.98	33.77
Lower lip area	<0.001	26.25	63.65*	85.74*	37.4	59.48*	22.09
Total lip area	<0.001	63.35	108.95*	164.81*	45.61	101.46*	55.86

Note

^*^ Indicates *P* value < 0.05 according to Tukey's post hoc test; N/S indicates “not statistically significant.” Mean difference of linear measurements is in mm; mean difference of angular measurements is in degrees; and mean difference of surface area measurements is in mm^2^.

Age‐related changes on all angle measurements were significant. Angles 1 and 2 increased from around 130 degrees to around 138 degrees from 20s to 50s, while angles 3 and 4 decreased from around 33 degrees to around 25 degrees (*P* < .001). The changes on these angles strongly manifested that the upper lip was gradually flattening with age.

To further analyze morphological changes and fully take the advantage of stereophotogrammetry, we measured the surface area of upper and lower lip. Upper lip surface area and lower lip surface area among women in their 20s were 478.44 and 467.51 mm^2^, respectively. In 50s age group, these numbers dropped to 399.37 and 381.77 mm^2^, respectively (*P* < .001). The significant age‐related lip surface area decrease indirectly demonstrated volume loss which made the lip look shriveled.

Notably, the intergroup comparison showed that age‐related changes on most anthropometric variables were subtle between 20s and 30s or between 30s and 40s and became more significant between 40s and 50s, indicating that lip aging accelerated after 40 among Chinese women.

**FIGURE 3:**
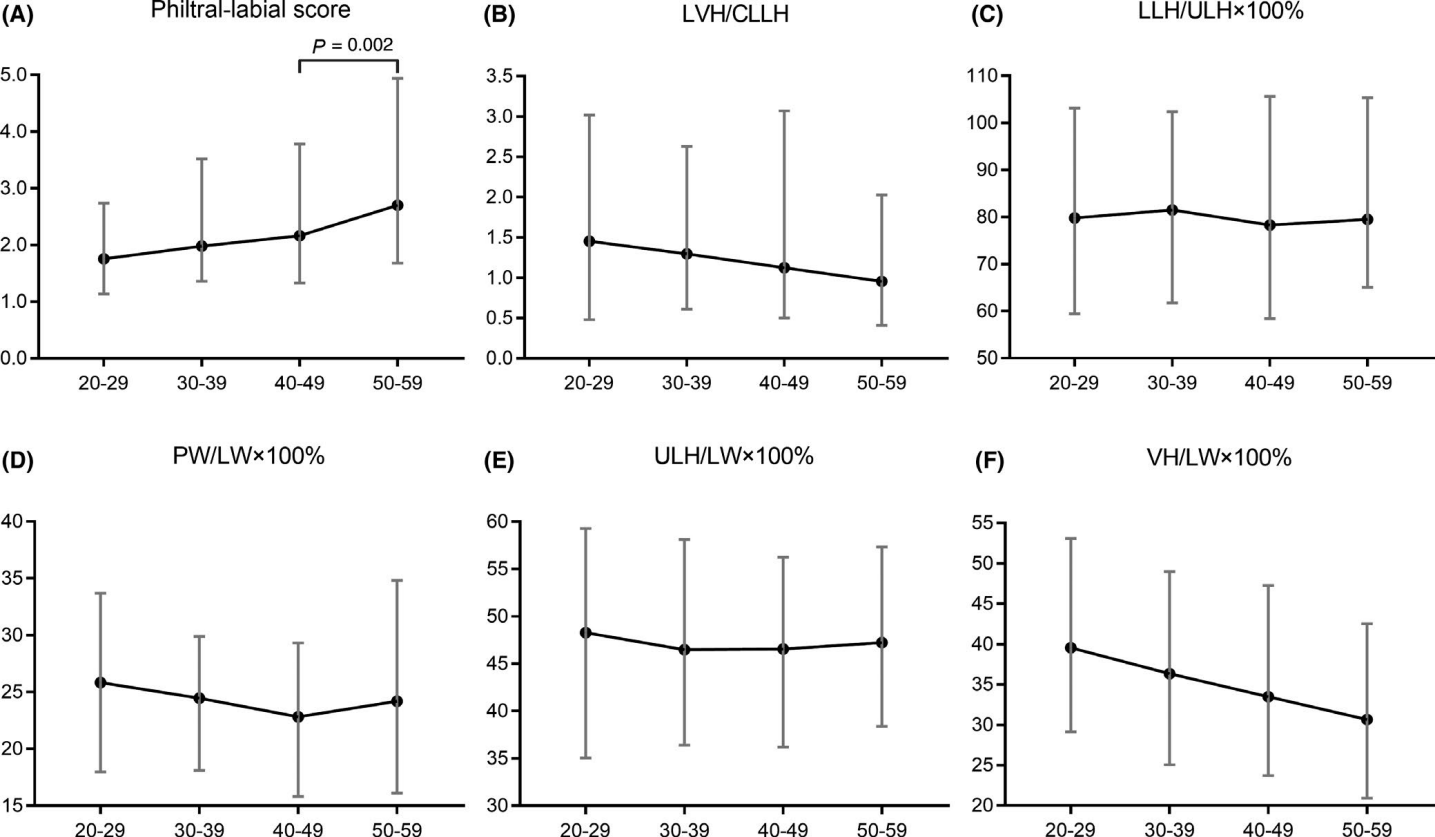
Mean and range of six labial proportions. (A) Philtral‐labial score; (B) LVH/CLLH; (C) LLH/ULH × 100%; (D) PW/LW × 100%; (E) ULH/LW × 100%; (F) VH/LW × 100%

Figure 3 showed anthropometric labial proportions of four age groups. Philtral‐labial scale, as an important indicator of upper lip morphology, increased significantly from 1.76 among 20s straight to 2.70 among 50s. Significant age‐related changes were also observed in sto‐li/li‐sl and ls‐li/ch‐ch, while the remaining three proportions showed no age‐related difference.

## 4 DISCUSSION

Of great aesthetic value, the lip is an important structure in determining one's attractiveness. The aging lip brings a frustrated and lifeless look, thus becomes a main target for facial rejuvenation. Many attempts have been made to lip rejuvenation by, for example, dermal filler injection, botulinum toxin application, and lip lift.^15^, ^16^ When giving these treatment, many surgeons lack objective numeric reference and relay on subjective impressive, which could lead to unproper correction.^17^ For better application of aesthetic procedures, anthropometric data should be collected for diagnosis of deformity, assessment of aging, and planning for surgical or nonsurgical procedures. Unlike many similar studies focusing on age‐related morphological changes which compared a young group with an elderly group several decades older, this study was designed to include several consecutive age groups covering Chinese women from 20 to 60. This could not only depict a continuous process of lip aging but provide data reference to reverse lip aging in women at different time of life.

Many attempts have been given to find anthropometric evidence on aging upper lip. Raschke et al used indexes to report relatively smaller upper vermilion height and larger cutaneous upper lip height in a large‐scale study on Caucasian males.^5^ Doll et al measured 45 women from northern Europe on 2D photographs and gave the similar conclusion.^4^ The cutaneous upper lip height significantly increased while the reduction of upper vermilion showed no statistical significance between subjects under 35 and over 50. In this Chinese women study, we found that the cutaneous upper lip elongation and upper vermilion height decrease were both significant in the comparison of 20s to 50s. Interestingly, when comparing two consecutive age groups, we noticed statistical significance was achieved only between 40s and 50s, not between 20s and 30s or 30s and 40s, indicating that upper lip morphological changes in the vertical dimension began to be noticeable after 40 among Chinese women. Furthermore, this study firstly evaluated how aging process affected the four angles related to the border of upper lip. The increase of angles 1 and 2 along with the decrease of angles 3 and 4 demonstrated that the vermilion border got increasingly flatten with age, giving an impression of less feminization.^16^ Bisson et al have examined these angles in their study comparing models from fashion magazine to ordinary people.^18^ They found the right and left bow angles (angles 1 and 2 in this study) were smaller in models and that the upper lip angle (angle 3 in this study) was larger in models. Results of their study and ours confirmed the instinct that aging process made the upper lip less aesthetically pleasing.

With regard to lower lip, age‐related changes were similar . The lower vermilion shortened with age while cutaneous lower lip height increased. These changes showed no statistical significance in any of the comparison between two consecutive age groups. Comparing to the age‐related shortening of upper vermilion, lower vermilion height decrease was less prominent, which was consistent with the results of previous studies.^4^, ^19^

Labial volume measurement has been proven to be difficult and possibly inaccurate because of the difficulty to demarcate boundaries on anthropometric analytical software.^17^, ^20^ In this study, we measured the surface area to indirectly predict age‐related volume change. We reported that both upper and lower vermilion surface area decreased significantly with age. The age‐related changes were statistically insignificant between any of the two consecutive age groups, indicating that the surface area decrease was a slow and gradual process. Measurement of vermilion volume was not new, but the results varied a lot on different study population. Our study showed that the average areas of vermilion among Chinese women in their 20s were 945.95 mm^2^, larger than what has been reported by Sawyer et al on Caucasian women aging from 21 to 49 (4.6 cm^2^)^17^ and by Ayoub *et al* on Lebanese women aging from 18 to 30 (422.69 mm^2^),^21^ smaller than the results from the study of Bisson et al (1284.08 mm^2^),^18^ but very close to the results reported by Ferrario et al and De Menezes et al^22^, ^23^

The attractive lip could be rebuilt by aesthetic procedures. Lip lift is widely used to reverse aging upper lip by making PLS ideally within 1.2 to 2.3.^24^ However, when it comes to give lip lift to Chinese women, the ideal PLS should be <2, because it seemed that PLS of Chinese women younger than 40 was under 2 (Figure 3A). Meantime, hydraulic acid is widely used for lip augmentation that can last for 4‐8 months. Reestablishment of a fine vermilion border is the key for aesthetic purposes, so the injection is often given along the vermilion border at the red roll.^25^ These surgical or nonsurgical procedures should not be done without orientation value. This cross‐sectional anthropometric analysis aims to deepen the understanding of how aging process affects labial morphology to help with pre‐ and post‐operative assessment so that under or over correction of aging lips could be avoid.

This is a stereophotogrammetry study on aging lips, taking advantage of 3D photography, and measuring technology. In recent years, 3D has been increasingly applied in anthropometric studies due to its good reliability and high accuracy.^26^ In the authors' opinion, 3D photography is especially suitable for labial morphology analysis where surface area difference and volume loss are important variables. Stereophotogrammetry could further show its strength in the evaluation of lip rejuvenation. Future study is on the way to validate the assessment of lip cosmetic procedures via 3D anthropometry.

One limitation is that the study population comes from 12 provinces of Northern China. The results could not represent the morphological characteristics of the lip of Southern Chinese women. Future study is expected to focus on Southern Chinese population and the North‐South differences in labial morphology.

## 5 CONCLUSION

This study provided baseline anthropometric data for lip morphology and demonstrated age‐related changes in Chinese women across four consecutive age groups. The lip tended to widen and extended in the vertical dimension. The vertical elongation was due to increase of cutaneous upper and lower lip height. Upper vermilion decreased in height and got increasingly flatten with age. These changes were significant after 40 years old. Aging process also brought significant surface area decrease to both upper and lower vermilion.
